# Analyzing how neuronal parameters influence network activity

**DOI:** 10.1186/1471-2202-12-S1-P26

**Published:** 2011-07-18

**Authors:** Anca Doloc-Mihu, Ronald L Calabrese

**Affiliations:** 1 Department of Biology, Emory University, Atlanta, GA 30322, USA

## 

A half-center oscillator (HCO) is a common circuit building block of central pattern generator (CPG) networks that produce rhythmic motor patterns in animals. Rhythmic activity in the heartbeat CPG of the leech is based on alternating bursting in two pairs of inhibitory interneurons that make reciprocal spike-mediated and graded synapses across the ganglionic midline. Our study here constitutes the next step toward a full investigation of how intrinsic membrane and synaptic parameters affect the electrical activity of a half-center oscillator (HCO) model and how different parameter regimes influence stability and modulatability of the HCO model’s output.

In our previous study [2], we constructed an efficient relational database table with the resulting characteristics of Hill et al.’s [1] HCO single-compartment conductance-based model. The model consists of two reciprocally inhibitory neurons and replicates the electrical activity of the oscillator interneurons of the leech heartbeat CPG under a variety of experimental conditions. To systematically explore the parameter space of this model, we used a brute-force approach. We varied a set of eight selected parameters (maximal conductances of intrinsic and synaptic currents) in all combinations resulting in a parameter space of 10,485,760 simulated models (10,321,920 HCO models and 163,840 corresponding isolated neuron models). After performing all the simulations, we built a SQL database table for their firing characteristics [3,4], which we can use to ask fundamental questions about the activity of HCOs.

We began our analysis by classifying these HCO and isolated neuron model simulations by their activity characteristics: models showing the same electrical activity are segregated to the same group. The HCOs were split into ten groups labeled: ***spiking***, ***silent***, ***asymmetric activity***, ***plateau*, *irregular spikes*, *asymmetric bursting*, *one burst*, *irregular period*, *unbalanced***, and ***functional***; the isolated neuron models were split into seven groups: ***spiking***, ***silent***, ***bistable***, ***plateau***, ***irregular spikes***, ***irregular period***, and ***regular bursting***[2]. By querying the database, we compared the activity characteristics of the groups of our simulated HCO models with those of our simulated isolated neuron models and found that ***regularly bursting*** neurons compose only a small minority of ***functional*** HCO models (419 models); the vast majority was composed of ***spiking*** neurons (32,568 models). This finding indicates that, within the parameter space considered, ***spiking*** neurons are highly prevalent, suggesting that this mode of activity is robust, and that ***regular bursting*** neurons are rare, suggesting that this state requires a delicate balance of intrinsic membrane conductances (parameters) and that they may not be robust to parameter changes. Also, our data suggest that ***regular bursting*** isolated neurons form ***functional*** HCOs that are more robust to variations in synaptic parameters than those formed by ***spiking*** isolated neurons.

We will now use the entire database to explore in depth the interaction of parameters that lead to the different activity groups we have identified. We will be particularly interested in parameter changes which correspond to known neuromodulations such as the modulation of h current by myomodulin [5].
